# A Higher Ovarian Response after Stimulation for IVF Is Related to a Higher Number of Euploid Embryos

**DOI:** 10.1155/2017/5637923

**Published:** 2017-03-27

**Authors:** Elena Labarta, Ernesto Bosch, Amparo Mercader, Pilar Alamá, Emilia Mateu, Antonio Pellicer

**Affiliations:** ^1^Human Reproduction Unit, Instituto Valenciano de Infertilidad (IVI), Plaza de la Policía Local 3, 46015 Valencia, Spain; ^2^PGD Laboratory, Instituto Valenciano de Infertilidad (IVI), Plaza de la Policía Local 3, 46015 Valencia, Spain

## Abstract

This study has analysed the relationship between ovarian response and the number of euploid embryos. This is a post hoc analysis of a subset of data generated during a prospective cohort study previously published. Forty-six oocyte donors were subjected to ovarian stimulation with 150 IU of rFSH and 75 IU of hp-hMG in a GnRH agonist long protocol. Preimplantation genetic screening was performed in all viable embryos. We observed a positive relationship between ovarian response and the number of euploid embryos. When ovarian response was above the median (≥17 oocytes), the mean number of euploid embryos per donor was 5.0 ± 2.4, while when <17 oocytes were obtained the mean number of euploid embryos was 2.7 ± 1.4 (*p* = 0.000). Aneuploidy rate did not increase with ovarian response or gonadotropin doses. Also, the number of euploid embryos was inversely related to the amount of gonadotropins needed per oocyte obtained (ovarian sensitivity index). These results suggest that the number of euploid embryos available for embryo transfer increases as the number of oocytes obtained does. Considering the total number of euploid embryos seems more relevant than the aneuploidy rate.

## 1. Introduction

The relatively high aneuploidy rate observed in human embryos after an IVF/ICSI cycle has been classically attributed to the technique itself, assuming that this prevalence might be lower in natural conceptions. Two main hypotheses have been proposed to contribute to these findings: (1) exogenous factors related to the IVF technique, such as controlled ovarian stimulation (COS) treatments or lab conditions; (2) a high ovarian response after using gonadotropins.

Ovarian stimulation effects have been well characterised in animals, mainly in the murine model, and have shown that aggressive stimulation leads to a poorer embryo development potential and could increase the chromosomal abnormalities rate [1, 2]. Yet in humans, studies are scarce and less conclusive. In this context when comparing natural and stimulated cycles, no differences have been observed in terms of cleavage capacity [3], oocyte [4], and embryo aneuploidy rate [5], or incidence of aneuploidy in either aborted foetuses [6] or chorionic villus sampling late in the first trimester of pregnancy [7].

Regarding high ovarian response, preliminary studies have suggested that it could be a determining factor in the genesis of aneuploidies, more than the gonadotropin doses used. It was suggested that women with a higher cohort of oocytes display more diploid oocytes and more cytoplasmic immaturity than those with a moderate or mild ovarian response [8], regardless of the doses used. More recent studies that have performed polar body analyses have shown that ovarian response to gonadotropins is positively related to oocyte aneuploidy and that the proportion of euploid oocytes is directly related to the number of MII oocytes and inversely related to the number of units of FSH per oocyte and per MII oocyte obtained [9, 10].

A positive relationship between ovarian response and the embryo aneuploidy rate has been described when mild COS is performed in infertile patients, but this is not observed in patients who undergo conventional COS treatments with higher doses [11]. It has been recently suggested that a threshold level for gonadotropin doses may exist and that no more competent oocytes can be obtained if it is exceeded [12].

Conversely, however, other recent studies have indicated that high ovarian response to gonadotropins is not so detrimental to embryo quality. In fact, it has been shown that high ovarian response to conventional ovarian stimulation does not increase embryo aneuploidy rates in aCGH-PGS cycles [13]. Besides, the larger the number of available euploid blastocysts, the higher the clinical pregnancy rate observed [14, 15].

One key point is to consider not only gonadotropin dose or ovarian response separately, but also their combination. This has been addressed in the “ovarian sensitivity index” (OSI) concept. A high OSI means that more oocytes with fewer gonadotropin doses are retrieved and offers the best pregnancy outcome, probably because it reflects a healthy ovary condition [16].

In the present study, our purpose was to analyse if women with a higher response to the same starting dose of gonadotropins for COS generate more euploid embryos than those with a lower response. Secondly, we investigated whether the necessity of more gonadotropin doses per egg obtained leads to fewer euploid embryos from the total cohort given.

## 2. Material and Methods

This study is a post hoc analysis of a subset of data generated during a prospective cohort study carried out in a university affiliated infertility clinic between September 2006 and March 2010 and has been previously reported [5]. Approval was obtained from the Institutional Review Board and the Institution's Ethics Committee prior to starting the study.

### 2.1. Study Population

The study was performed within our oocyte donation programme in couples for which a sperm donation was also required. Therefore, only the gametes from young subjects free of an infertility background were considered. The inclusion and exclusion criteria are described elsewhere [5].

### 2.2. Study Design

Forty-six oocyte donors underwent a GnRH agonist long protocol with a combination of 150 IU of recFSH (Gonal F®, Merck-Serono, Geneva, Switzerland) and 75 IU of highly purified hMG (Menopur®, Ferring Pharmaceuticals, Copenhagen, Denmark). rCG was administered when ≥6 follicles were >17 mm in diameter. Doses were adjusted according to ovarian response. Ultrasound-guided transvaginal oocyte retrieval was scheduled 36 h after triggering ovulation.

ICSI was performed in all cases by following our routine practice in PGS cycles. Fertilisation was assessed after 17–20 h and embryo cleavage was recorded every 24 h. Embryos were grown in IVF/CCM medium (I/I) (Vitrolife®, Göteborg, Sweden) until day 3 (d3) and were subsequently cultured in CCM medium with a monolayer of endometrial epithelial cells until d5 [17].

Embryo biopsy was performed on d3 in a noncontact laser system (OCTAX, Herbron, Germany). Only those embryos with ≥5 nucleated blastomeres of similar size and with a fragmentation degree of <20% were biopsied. A single blastomere was removed and a fluorescence in situ hybridisation (FISH) analysis was performed for nine chromosomes in two consecutive hybridisation rounds: 1st MultiVysion™ PB panel for chromosomes 13, 16, 18, 21, and 22; 2nd MultiVysion 4 Color Custom panel for chromosomes 15, 17, X, and Y (Vysis®, Inc. Downers Grove, IL, USA). Additional hybridisation rounds for these chromosomes, using probes that bind to different loci, were conducted to rescue the nonconclusive results and to confirm certain aneuploidies [18].

*Statistical Analysis.* The population with a high and a low response to ovarian stimulation was defined around the median (50th percentile, P50) once normal ovarian response distribution had been proved. A comparison between both groups was made by Student's *t*-test for independent groups for quantitative variables and by the Chi-square for qualitative variables. A *p* level of <0.05 was considered statistically significant. The relationship among the quantitative variables was analysed by a linear regression, and a statistical analysis was performed using the Statistical Package for the Social Sciences 21.0 (SPSS Inc., Chicago, IL, USA).

### 2.3. Outcome Measures

The primary objective was to analyse if women who present a high response to COS (considered to be the number of oocytes ≥ median) produce more euploid embryos than those with a lower response.

The secondary objectives were as follows:

- Correlation between ovarian response and (i) number of euploid embryos; (ii) aneuploidy rate; (iii) euploidy rate; (iv) cumulated pregnancy outcome.
- Correlation between total administered gonadotropin doses and the aneuploidies/euploidy rate.
- Correlation between OSI and the number of euploid embryos and the aneuploidies rate.

## 3. Results

The mean age of oocyte donors was 25.4 ± 4.0 years. Their BMI was 22.5 ± 2.8 (kg/m2) and basal FSH and E2 levels were 6.1 ± 2.1 IU/L and 48 ± 15 pg/L, respectively. The descriptive data of the follicular phase parameters and embryo development are outlined in Table 1.

The median (or percentile 50) number of oocytes retrieved was 17. Twenty-two women produced less than 17 oocytes, whereas the remaining 24 produced 17 oocytes or more. Age and ovarian stimulation parameters according to this cut-off were similar (Table 2).

The mean number of euploid embryos per oocyte donation was 3.9 ± 2.3 (95% CI: 3.2–4.6), with a minimum of 0 and a maximum of 10 euploid embryos per cycle (P25 to P75 = 2–5 euploid embryos). A total of 3.4 MII oocytes were needed to obtain one euploid embryo. The mean number of euploid embryos per cycle was closely related to ovarian response. In fact, when we considered the median value as a reference point, we observed that the mean of euploid embryos was 5.0 ± 2.4 (median 5.0) when the number of oocytes obtained was ≥median (17 oocytes), and it was 2.6 ± 1.4 (median 2.5) when ovarian response was below it (*p* < 0.001).

**Figure 1:**
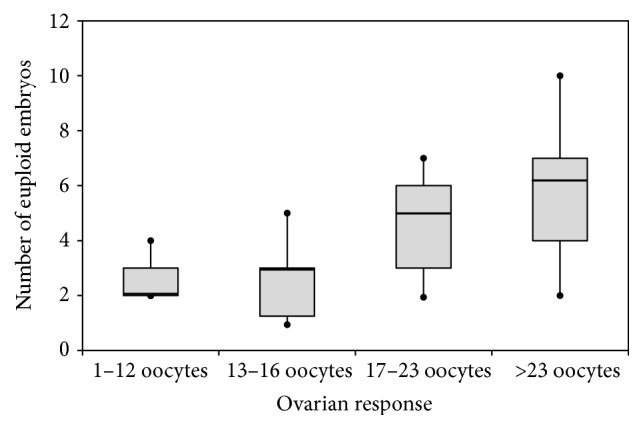
Number of euploid embryos according to ovarian response.

We observed a positive relationship between number of oocytes and number of euploid embryos (*p* < 0.05) (Figure 1). A linear regression evaluating this relationship provided the following equation: number of euploid embryos = 0.841 + 0.168 × number of oocytes, with *R* = 0.550; *R*^2^ = 0.302; *p* < 0.0001.

**Figure 2:**
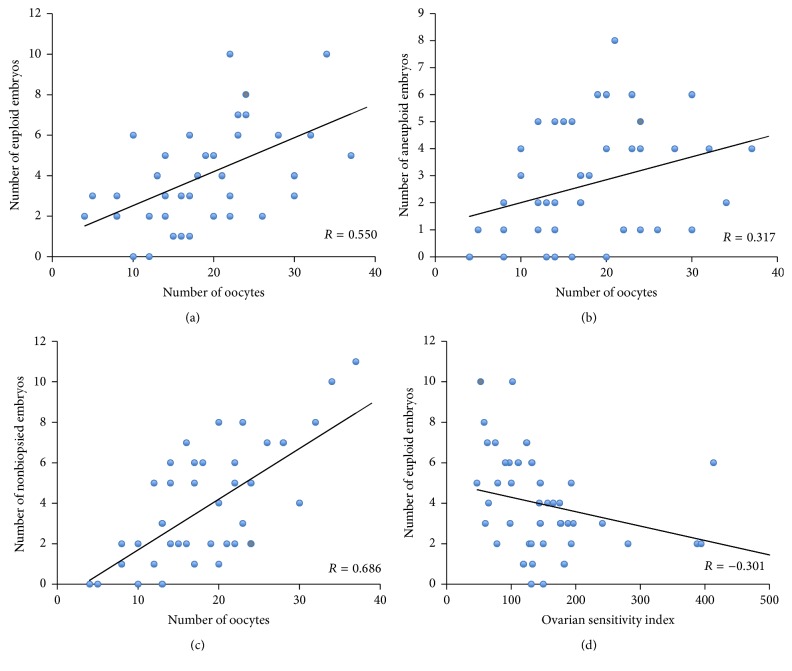
Correlation between the number of total oocytes obtained and the total number of euploid (a), aneuploid (b), and nonbiopsied (c) embryos per donor. Correlation between ovarian sensitivity index (OSI) and euploid embryos (d).

Ovarian response also correlated positively with the number of aneuploid and nonbiopsied embryos (e.g., arrested or not and meeting the inclusion criteria for being biopsied) (*p* < 0.05) (Figures 2(b) and 2(c)).

The number of euploid embryos was negatively related to the OSI (*p* = 0.04), which indicates that the women who produced more oocytes in response to lower gonadotropin doses (low OSI) were those who displayed more euploid embryos which, therefore, reflects better ovarian competence (Figure 2(d)).

**Figure 3:**
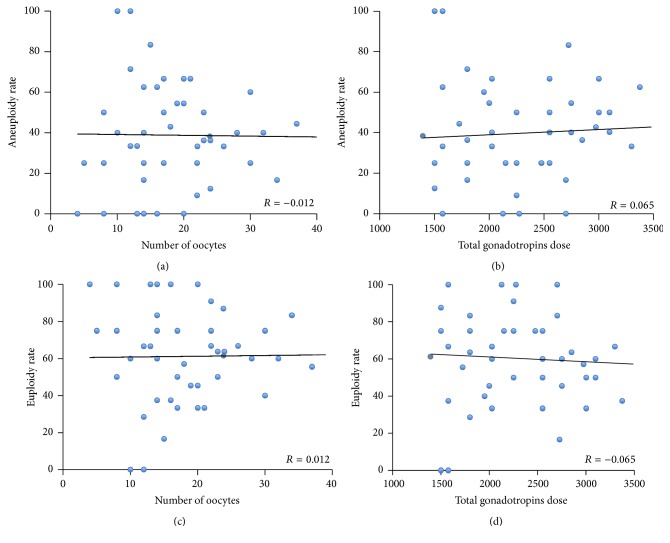
Correlation between ovarian response and gonadotropins doses and the aneuploidy rate (a and b) and euploidy rate (c and d), respectively.

Moreover, the aneuploidy rate was not dependent on ovarian response or gonadotropin dose (Figures 3(a) and 3(b), resp.). Similarly, the euploidy rate was not dependent on ovarian response or gonadotropin dose (Figure 3).

The cumulative live birth rate according to being above or below the median of the total number of oocytes obtained was 70.8% versus 50.0% (*p* = 0.15), respectively.

## 4. Discussion

The present study showed in a population of young normovulatory women that a high ovarian response after COS with moderate gonadotropin doses did not increase the embryo aneuploidy rate. Indeed, the higher the ovarian response, the more the euploid embryos obtained as a linear positive relationship was observed between both events. When ovarian response was above the median, the number of euploid embryos is double that when ovarian response was below it. Consequently, the cumulative live birth rate tended to be higher in these cases but did not reach statistical significance, as the study was not powered for this endpoint. Moreover, the present study shows that, in this population, a high response does not compromise the quality of the oocytes, as the proportion of euploid embryos obtained per each oocyte collected was similar to that one observed in the lower response.

The relationship between ovarian response and cycle outcome has been matter of debate for more than two decades. Large population studies have revealed that above a certain oocyte yield threshold, typically around 15 [19], no benefit in live birth rate terms is obtained. Whether this effect is due to endometrial impairment or to harm to the oocyte cohort remains controversial. Some authors have suggested that the capability of producing euploid and viable embryos of the women who undergo IVF is poor and limited (around 2 per cycle) [11]. In this context, a recent study has been unable to find a linear relationship between FSH doses and the overall number of blastocysts or by AMH strata. Thus a threshold level may exist for starting the gonadotropin dose, and no more competent oocytes can be obtained if it is exceeded [12].

Based on this hypothesis, softer ovarian stimulation protocols have been claimed since, according to this concept, obtaining a larger number of oocytes would be useless. Ovarian stimulation must meet the condition of achieving the highest possible degree of efficiency with the lowest risk and best cost-effectiveness. This increasingly accepted reality, along with growing evidence that high levels of steroid hormones during ovarian stimulation may affect oogenesis, the quality and development of the embryo [20], and endometrial receptivity [21], has led to a group that advocates the advantages of “mild ovarian stimulation” [22, 23]. Its objectives can be easily entered; they emphasise the best result with the least possible risk and cost and argue that the ultimate goal of these treatments is the birth of a single healthy child and thus avoids the dreaded risk of developing ovarian hyperstimulation syndrome (OHSS), an event that is likely to threaten the patient's life.

However, recent studies have not supported the concept that ovarian stimulation is so detrimental. An analysis of cumulative ongoing and live birth rates has shown that there is a dose-dependent relationship between ovarian response and cycle outcome because there are more chances of transferring remnant embryos in frozen-thawed cycles [24, 25]. This may increase the overall IVF pregnancy chance per oocyte pick-up by approximately 10–15% [26]. Other authors have been unable to find a negative relationship between high ovarian response and implantation rates or proportion of high quality embryos and have concluded that oocyte quality is not affected by ovarian response [27].

Studies in human oocytes [9, 10] have suggested that obtaining more MII oocytes in response to moderate gonadotropin doses entails a lower risk of aneuploidy and reflect adequate ovarian health. Those follicles that can be recruited after a minimum stimulus with exogenous gonadotropins are more likely to generate competent oocytes [28]. This agrees with a study by our group performed in oocyte donors, in which if gonadotropin doses were lower than those administered in a previous cycle in the same donor and the same number of oocytes in the ulterior cycle was obtained, the embryo aneuploidy rate lowered [29].

This might be explained by the recent concept of ovarian sensitivity index (OSI: dose of gonadotropins per obtained oocyte), which has been related to pregnancy rate: the smaller the amount of gonadotropins administered per obtained oocyte, the higher the pregnancy rate [16]. In the present study, we have observed that while gonadotropin doses are not positively related to the aneuploidy rate, OSI is negatively related to the number of euploid embryos. This means that the higher the ovarian response with a small amount of gonadotropins, the larger the number of euploid embryos obtained. This is also supported by the results published in a meta-analysis, which investigated the relationship between ovarian response according to the gonadotropin doses administered and pregnancy rates [30]. It revealed that the best implantation rates were obtained with 5 oocytes after mild COS due to smoother follicles selection, whereas 10 oocytes were needed after conventional COS. It concluded that poor oocyte yield after classical COS implies ovarian ageing.

These data reinforce the idea that the quality of the oocyte and embryo cohort depends on ovarian response to a given gonadotropin dose. Ovarian response in oocyte donors is considerably higher than that observed in infertile patients because the former are younger and have a higher ovarian reserve. In fact, high ovarian response with a median of 17 oocytes was obtained in our oocyte donors after taking into account the fact that the mean gonadotropin dose per obtained oocyte was 157 ± 100 IU daily. However in the review of Verberg et al. [30], an approximate mean of 200 IU was used in infertile patients per obtained oocyte, regardless of the protocol used (mild or conventional). We suggest that the reproductive potential of the embryo cohort produced by a young donor is closely related to ovarian response, which reflects healthy ovarian competence in a moderate COS context. It remains unknown whether these women receiving a higher dose of gonadotropins would have produced more euploid embryos. In this context, findings in fertile patients subjected to PGS for gender selection suggest a positive association between increasing total gonadotropin dose for COS and improving proportions of available euploid embryos for transfer [15].

There is increasing evidence that demonstrates that the number of euploid embryos correlates positively with the number of total oocytes, similarly to our findings. It has been evidenced with not only mild or moderate doses, but also with conventional stimulation, that high ovarian response does not increase embryo aneuploidy rates in an aCGH-PGS cycle context, both in infertile patients and in oocyte donors [13]. The aneuploidy rate is constant regardless of the number of biopsiable embryos available; the chance of having at least one euploid embryo increases, as does the number of biopsiable embryos. The above author's results indirectly corroborate that women with high ovarian reserve are more prone to live births.

This is consistent with the present study, in which we observed that when ovarian response was above the median, cumulative pregnancy rates were higher as more euploid embryos were available for transfer. These findings have been also demonstrated recently in infertile patients. In fact, in women with ≥4 euploid blastocyst availability, a single embryo transfer offers comparable pregnancy rates to a double embryo transfer, while multiple pregnancy rates significantly lower. In women with ≤3 euploid blastocysts, clinical pregnancy rates were higher when a double embryo transfer was performed [14]. These findings support the hypothesis that euploid embryo cohort size is a prognosis factor in IVF treatments and that the number of euploid embryos has to be taken into account, more importantly than the percentage of euploid/aneuploid embryos, as a prognosis factor, especially if we consider cumulative pregnancy outcome [31]. In fact, according to the results of our study, both the aneuploidy and euploidy rates do not vary according to ovarian response, whereas the number of euploid and aneuploid embryos increases in the same way as number of oocytes does. This means that higher ovarian response increases both euploid and aneuploid embryos number but the proportion between them is not altered, so it is not detrimental for embryo quality.

The main limitation of our study is that at the time it was designed and conducted, the FISH method was used for PGS and did not allow a comprehensive chromosomal analysis. Embryo biopsy was performed on d3, although we had previously demonstrated a high correlation between the results obtained on d3 or d5 after biopsy even though the array-CGH protocol for PGS is used [32]. It should also be noted that nowadays our donors are stimulated according to a GnRH antagonist protocol and undergo GnRH triggering in all cases to avoid ovarian hyperstimulation syndrome (OHSS). It has to be considered that our study population exclusively included young fertile women, so extrapolating our findings to the most frequently treated old infertile population should be done cautiously. As previously mentioned, however, recent results support our findings even in this last situation. Although sample size is limited for a retrospective analysis, we consider that with such significant differences between the groups (above and below the median) the probability that it is an incidental finding is marginal. The strength of our study is that the results come from a prospective design in which all donors included fulfilled strict inclusion criteria and were stimulated with the same protocol, receiving same initial doses of gonadotropins. The fact that they initially received the same doses of gonadotropins allows us to hypothesize that, with similar doses, a higher response leads to a higher number of good quality embryos. The interesting question is whether trying to increase the ovarian response through an increase in doses of gonadotropins could generate more euploid embryos or whether in that case we would obtain only aneuploid ones. Our group published a study in oocyte donors addressing this issue in which it was observed that if a higher ovarian response after receiving significant higher doses of gonadotropins was achieved, the number of euploid embryos per MII was significantly lower (*p* = 0.02). On the other hand, if the response was similar regardless of the dose, this difference turned to be nonstatistically significant. This reinforces the idea that ovarian response in terms of number of oocytes obtained is not detrimental for the results of the cycle, as long as the lowest as possible doses of gonadotropins are given.

In conclusion, we observed that the more oocytes retrieved, the more euploid embryos are obtained. Therefore, we hypothesize that the cumulative probability of achieving ongoing pregnancy in an oocyte donation setting could be greater. The remaining question is whether this can also be extrapolated to infertile patients with good ovarian reserve. In this population, the key point is to obtain a good number of oocytes with smaller amounts of the needed gonadotropins (low OSI), and avoiding the risk of OHSS should always be taken into account and attempted. Moreover, high ovarian response is associated with elevated progesterone at the end of the follicular phase, which is known to impair embryo implantation due to a less receptive endometrium [21]. If these events occur, safe alternatives such as GnRH triggering, and oocyte or embryo vitrification strategies, are a real option.

## Figures and Tables

**Table 1:** Follicular phase and embryo development parameters in 46 oocyte donors subjected to COS.

Cycle parameters	
Duration of stimulation (days)	10.1 ± 2.3
Total gonadotropin doses (IU)	2230 ± 670
Serum E2 on rCG day (pg/ml)	2707 ± 1135
Serum P on rCG day (ng/ml)	0.8 ± 0.4
Number of oocytes	17.8 ± 7.7
Number of metaphase II oocytes	13.4 ± 6.2
Number of oocytes fertilised	10.2 ± 4.8
Number of biopsied embryos	6.7 ± 3.5
Number of informative embryos	6.6 ± 3.4
Aneuploidy rate in the whole embryo cohort	40.6% (95% CI: 35.0–46.4)
Mean aneuploidy rate per egg donor	38.2% (95% CI: 30.5–45.8)

Plus-minus values are mean ± SD.

**Table 2:** Comparison of the different parameters between women with ovarian response below the median (<17 oocytes) or equal to/above the median (≥17 oocytes).

	Ovarian response < median (<17 oocytes) (*n* = 22)	Ovarian response ≥ median (≥17 oocytes) (*n* = 24)	*p* value
Age	25.6 ± 4.21	24.9 ± 4.0	0.556
Days of stimulation	9.6 ± 2.0	10.5 ± 2.5	0.161
Total doses of FSH (IU)	1579 ± 480	1633 ± 513	0.715
Total doses of hp-hMG (IU)	721 ± 289	653 ± 265	0.413
Serum E2 on hCG day (pg/ml)	2622 ± 1157	2808 ± 1150	0.592
Serum P4 on hCG day (ng/ml)	0.70 ± 0.38	0.89 ± 0.30	0.078
Mean of euploid embryos	2.7 ± 1.4	5.0 ± 2.4	0.000
Number of oocytes obtained	11.5 ± 3.4	23.8 ± 5.5	0.000
Number of embryos biopsied	4.5 ± 2.3	8.3 ± 3.3	0.000
Number of euploid embryos/embryo biopsied	0.61 ± 0.31	0.61 ± 0.18	0.963
Number of euploid embryos/oocyte obtained	0.25 ± 0.16	0.22 ± 0.10	0.422

Plus-minus values are mean ± SD.
