# Corrigendum to “A Higher Ovarian Response after Stimulation for IVF Is Related to a Higher Number of Euploid Embryos”

**DOI:** 10.1155/2017/1718068

**Published:** 2017-07-13

**Authors:** Elena Labarta, Ernesto Bosch, Amparo Mercader, Pilar Alamá, Emilia Mateu, Antonio Pellicer

**Affiliations:** ^1^Human Reproduction Unit, Instituto Valenciano de Infertilidad (IVI), Plaza de la Policía Local 3, 46015 Valencia, Spain; ^2^PGD Laboratory, Instituto Valenciano de Infertilidad (IVI), Plaza de la Policía Local 3, 46015 Valencia, Spain

In the article titled “A Higher Ovarian Response after Stimulation for IVF Is Related to a Higher Number of Euploid Embryos” [1], an incorrect version of Figure 2(d) is published. The correct version is shown here.

Accordingly, in Results, the text reading “The number of euploid embryos was negatively related to the OSI (*p* = 0.04), which indicates that the women who produced more oocytes in response to lower gonadotropin doses (low OSI) were those who displayed more euploid” should be changed to “The number of euploid embryos was positively related to the OSI (*p* = 0.04), which indicates that the women who produced more oocytes in response to lower gonadotropin doses (high OSI) were those who displayed more euploid.”

Also, in Discussion, the text reading “This might be explained by the recent concept of ovarian sensitivity index (OSI: dose of gonadotropins per obtained oocyte), which has been related to pregnancy rate: the smaller the amount of gonadotropins administered per obtained oocyte, the higher the pregnancy rate [16]. In the present study, we have observed that while gonadotropin doses are not positively related to the aneuploidy rate, OSI is negatively related to the number of euploid embryos” should be changed to “This might be explained by the recent concept of ovarian sensitivity index (OSI: oocytes recovered × 1000/total dose of gonadotropins), which has been related to pregnancy rate: the smaller the amount of gonadotropins administered per obtained oocyte, the higher the pregnancy rate [16]. In the present study, we have observed that while gonadotropin doses are not positively related to the aneuploidy rate, OSI correlates positively to the number of euploid embryos.”

## Figures and Tables

**Figure 2:**
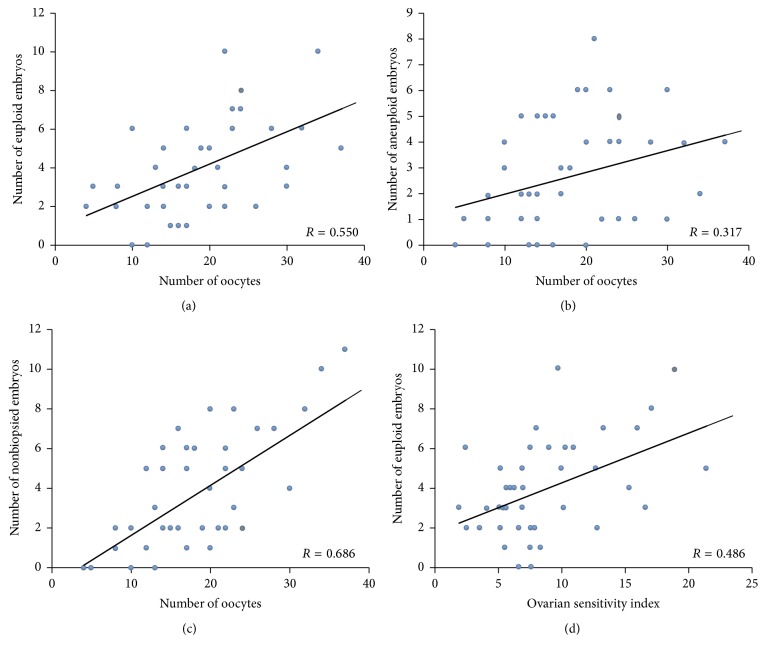
Correlation between the number of total oocytes obtained and the total number of euploid (a), aneuploid (b), and nonbiopsied (c) embryos per donor. Correlation between ovarian sensitivity index (OSI) and euploid embryos (d).
